# Factors associated with *Anaplasma phagocytophilum* PCR positivity and seropositivity in horses in Europe (2022–2025)

**DOI:** 10.1186/s13071-026-07770-x

**Published:** 2026-11-14

**Authors:** Sarah Grundmann, Christina Strube, Anna Sophie Hecker, Carla Wiebke Axt, Andreas Moritz, Elisabeth Müller, Ingo Schäfer

**Affiliations:** 1 Institute for Parasitology, Centre for Infection Medicine, University of Veterinary Medicine Hannover, Hannover, Germany; 2 Laboklin GmbH & Co. KG, Bad Kissingen, Germany; 3 Institute of Parasitology, Faculty of Veterinary Medicine, Leipzig University, Leipzig, Germany; 4 Small animal clinic – Internal Medicine, Department of Veterinary Medicine, Justus-Liebig-University, Giessen, Germany

**Keywords:** Granulocytic anaplasmosis, Equine granulocytic anaplasmosis, EGA, Ticks, Tick-borne diseases, Vector-borne diseases, Immunofluorescence antibody test, Antibodies, Polymerase chain reaction, One health

## Abstract

**Background:**

Equine granulocytic anaplasmosis (EGA) is caused by *Anaplasma phagocytophilum* and mainly transmitted by *Ixodes persulcatus* complex ticks, particularly *I. ricinus* in Europe. The study aimed to investigate factors associated with PCR positivity and seropositivity in horses.

**Methods:**

Equine samples from across Europe submitted to a laboratory between January 2022 and December 2025 were retrospectively analyzed. Polymerase chain reaction (PCR) results on EDTA blood and serology using immunofluorescence antibody test (IFAT) on serum or plasma were included. Multiple logistic regression analysis was performed, in which horses were grouped in different age categories.

**Results:**

Of 2,753 horses tested by PCR, 358 (13.0%) were positive. In the multivariable model, PCR positivity was associated with season, sampling year, European region, age category, sex, and horse type classification. The strongest seasonal effect was observed in winter, with lower odds compared to spring (OR=0.033, *P*<0.001). Foals and weanlings had higher odds than adult horses (OR=3.927, *P*=0.002), whereas ponies had lower odds than warmbloods (OR=0.502, *P*<0.001). Seropositivity was 26.0% (1201/4615 horses). Years of sampling, age category, and horse type were associated with seropositivity in the IFAT model. The highest odds were observed in 2025 compared to 2022 (OR=1.772, *P*<0.001), in senior horses >18 years (OR=1.640, *P*<0.001), and in heavy horses compared with warmbloods (OR=2.740, *P*<0.001).

**Conclusions:**

Descriptively, PCR-positivity was highest in summer and lowest in winter; adjusted pairwise comparisons confirmed significantly lower odds in winter compared with the other three seasons, in accordance with tick activity patterns. Further significant differences between different seasons were not noted. The increasing seropositivity with increasing age can be interpreted as higher risk for pathogen contact during life. Horse type-associated differences may reflect unmeasured differences in management, housing conditions, and vector exposure rather than intrinsic biological susceptibility. Given the multi-host ecology of *A. phagocytophilum* and the zoonotic potential of some genetic variants, equine infections are relevant from a One Health and All-species perspective.

**Graphical Abstract:**

**Figure:**
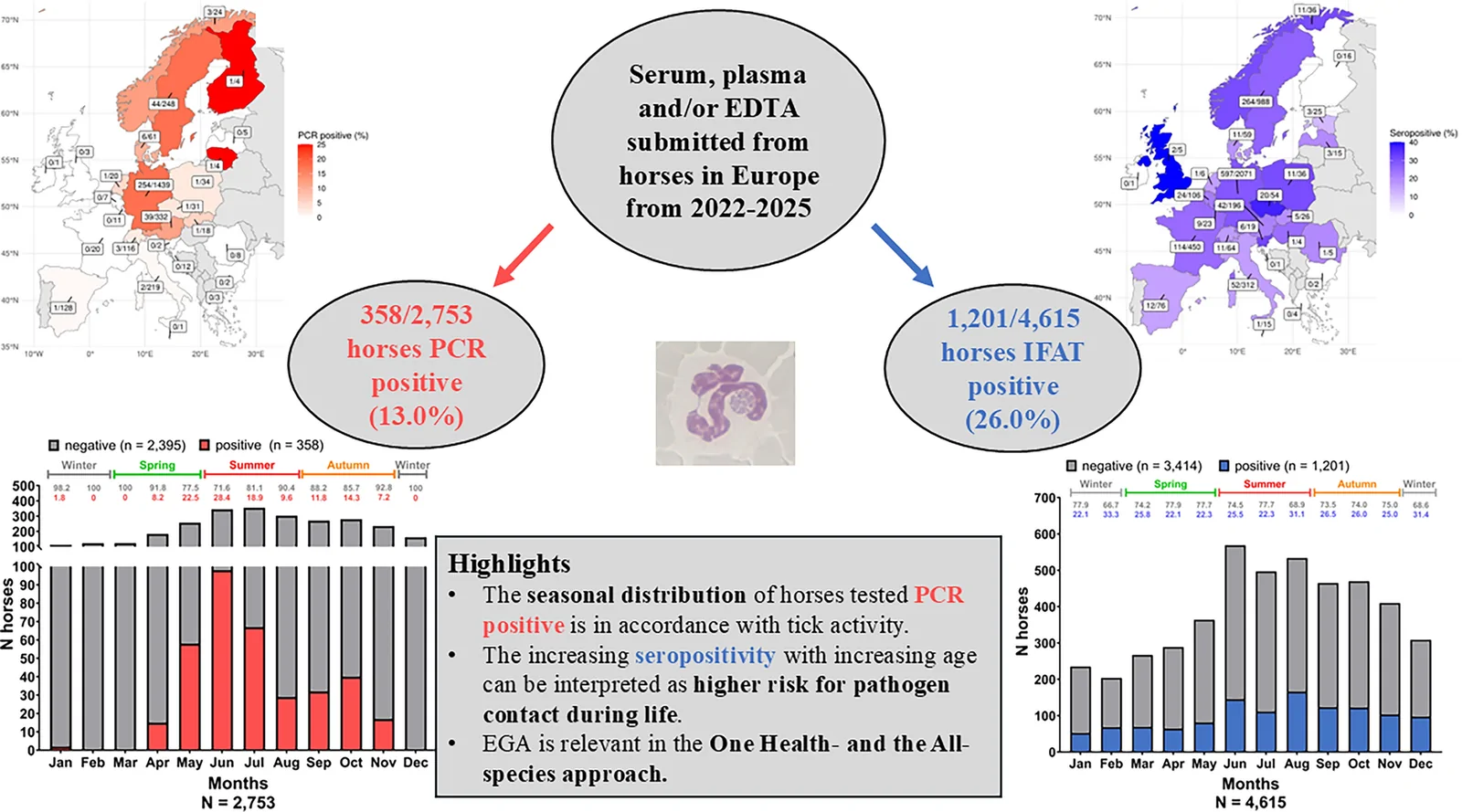

**Supplementary Information:**

The online version contains supplementary material available at https://doi.org/10.1186/s13071-026-07770-x.

## Background

Equine granulocytic anaplasmosis (EGA) is a vector-borne disease caused by the obligate intracellular, Gram-negative bacterium *Anaplasma phagocytophilum* [1]. *Anaplasma phagocytophilum* also causes infections in dogs [2], cats [3], ruminants [4], wildlife [5], and humans [6], placing it within the All-species approach and One-Health framework. Based on the *groEL* gene, four ecotypes (I–IV) with distinct host and vector associations are known. Ecotype I has the broadest host range and includes both zoonotic and non-zoonotic variants. Isolates from humans, horses, dogs, cats, and wildlife cluster within a specific zoonotic subgroup, while ruminant-associated variants are more abundant but rarely zoonotic [7]. Feline *A. phagocytophilum* strains are genetically indistinguishable from those causing disease in humans, dogs, and horses, although the clinical significance of this overlap is unclear, since granulocytic anaplasmosis is diagnosed far less often in cats [8]. In horses, molecular typing has revealed several distinct genetic variants (16S rRNA, *groEL*, *msp2*, *msp4*), with no clear association between genotype and host or disease severity [9].

Ticks of the *Ixodes persulcatus* complex, primarily *Ixodes ricinus* ticks in Europe, are the principal vectors [1]. Serological and/or molecular evidence of EGA has been reported across almost whole Europe. A broad north-to-south gradient is apparent, with reported prevalence differing considerably between diagnostic methods and countries or regions. Reported rates include 17–69% PCR positivity and 17% IFAT seroprevalence in Sweden [10, 11], 22% ELISA-based seroprevalence in Denmark [12], 10% PCR positivity in the Netherlands [13], 11.3% ELISA-based seroprevalence in southern France [14], 15% PCR positivity and 27% IFAT seroprevalence in Germany [15, 16], 5% PCR positivity and 5–73% IFAT seroprevalence in the Czech Republic and Slovakia [17–19], 1–5% PCR positivity in Poland/Slovakia/Ukraine [20], 4% IFAT seroprevalence in Switzerland [21], and further confirmed exposure by serology in Bulgaria [22] and Romania (10.3% seroprevalence) [23], several Italian regions (9–23% IFAT seroprevalence; PCR positivity generally lower where tested) [24–27], Spain (6.5% seroprevalence, Galicia) [28] and Greece (14% seroprevalence) [29]. In one study from Germany, most horses tested PCR-positive in summer, followed by spring, which is in accordance with the main activity of *I. ricinus* ticks [15]. Next to vectorial transmission, transplacental transmission of *A. phagocytophilum* after experimental infection [30] and infections through blood transfusion have been reported [31].

EGA ranges from subclinical infections to severe disease, with an incubation period from one to three weeks [32]. Horses under four years of age exhibit milder clinical signs [32]. EGA infections can be diagnosed in blood by PCR and/or by observing morulae in neutrophils, and rarely eosinophils, in peripheral blood smears and/or buffy coats [33, 34]. PCR is more sensitive [33, 34], as morulae can only be observed as intracellular inclusions in granulocytes two to four days after the onset of disease [30, 35]. Detection of *A. phagocytophilum* antibodies by IFAT on serum or plasma is mainly of epidemiological interest to provide information on pathogen contact in the past, but of limited clinical value due to the acute onset of EGA [31].

The pathophysiology of EGA remains poorly understood. Forest, water bodies, and landscape heterogeneity in Sweden were associated with higher likelihood of infections [11], with sex and proximity to swallow colonies/waterways in France [14], and with elevation above sea level in northern Italy [27]. Outside Europe, age, breed, season, and ectoparasite infestation contributed to infections in Egyptian horses [36], while sex and region had no impact in Tunisian horses [37]. This illustrates inconsistent findings across individual study populations. In cats, seroprevalence has been linked to housing/outdoor access [38], and testosterone is known to increase tick-borne transmission potential and ectoparasite burden in males of several species [39, 40]. Whether comparable factors shape EGA risk in European horses remains largely unknown.

The knowledge regarding the pathophysiology of EGA and its epidemiology in Europe is limited. Therefore, the aim of our study was to investigate potential influencing factors for EGA infections and seropositivity in horses, including signalment, regionality, seasonality, and annual patterns.

## Methods

### Diagnostics for detecting equine *A. phagocytophilum* infections

EDTA and serum/plasma samples were submitted by veterinarians across Europe to the laboratory Laboklin (Bad Kissingen, Germany) between January 2022 and December 2025 for routine diagnostics on a fee-for-service basis. Samples were sent by overnight shipment to the laboratory. This data was retrospectively analyzed, and includes both direct and indirect diagnostic methods for detection of *A. phagocytophilum* in equine samples. A TaqMan real-time PCR (Applied Biosystems/Life Technologies, target gene: 60-kDa heat shock protein [HSP60]) on EDTA blood was used for direct pathogen detection. A volume of 200 µL EDTA blood was processed for automated total nucleic acid isolation using a commercial kit (MagNA Pure 96 DNA and Viral NA Small Volume Kit, Roche Diagnostics GmbH, Mannheim, Germany), following the manufacturer’s instructions. The amplification protocol consisted of an initial denaturation at 95 °C for 30 s, followed by 40 cycles of denaturation at 95 °C for 5 s and annealing/extension at 60 °C for 30 s. Samples with Ct values below 35 were classified as positive. To ensure analytical reliability, each PCR run included negative and positive controls, as well as an extraction control (DNA Process Control Detection Kit, Roche Diagnostics GmbH, Mannheim, Germany) to monitor nucleic acid recovery and potential PCR inhibition.

IgG antibodies were assessed in serum or plasma using an IFAT (MegaFLUO ANAPLASMA, MegaCor Diagnostik, Hörbranz, Austria). Antibody titers > 1:40 were interpreted as positive.

### Potential risk factors and categorization

Information on sex, age, and breed of each horse, as well as the month and year of analysis was documented. Seasonality was categorized as follows: spring (March–May), summer (June–August), autumn (September–November), and winter (December–February). If multiple samples were submitted from the same horse, the first sample was used for statistical analysis. If this first sample tested negative but a later sample from the same horse tested positive, the first positive sample was used instead. For the regional distribution across Europe, countries were categorized as follows: Central (Austria, Germany, Switzerland), Western (Belgium, France, Luxembourg, Netherlands), Eastern (Czech Republic, Estonia, Hungary, Lithuania, Latvia, Poland, Romania, Slovakia), Northern (Denmark, Finland, United Kingdom, Ireland, Norway, Sweden), and Southern Europe (Bulgaria, Spain, Greece, Croatia, Italy, Malta, Slovenia). Horses with known breed were classified into four groups: warmblood horses, thoroughbreds, heavy horses, and ponies. Icelandic horses were assigned to the pony group because of comparable physical characteristics and similar husbandry conditions.

### Statistical analysis

Descriptive statistical analyses were conducted using SPSS for Windows (version 28.0; IBM), and model analyses were performed in R version 4.5.1 [41]. A *P*-value < 0.05 was considered statistically significant. Separate multivariable binary logistic regression models with a logit link were fitted for PCR and IFAT results, with each test outcome coded as negative or positive. Horses with missing predictor data were excluded from the respective model using complete-case analysis. Within each category of the model predictors, PCR- and IFAT-positivity proportions were compared between included and excluded horses using two-sided Fisher’s exact tests, with Holm adjustment performed separately for PCR and IFAT. During initial development of the conventional PCR logistic regression model, candidate predictors were added sequentially and evaluated according to their contribution to model fit. Model development was guided by likelihood-ratio tests and comparisons of Akaike’s information criterion (AIC) and Bayesian information criterion (BIC). The final model included season (spring, summer, autumn, winter), sampling year (2022, 2023, 2024, 2025), European region (Central, North, South, East, West), sex (intact male, gelding, female), horse type classification (warmblood, pony, thoroughbred, heavy horse), and age category as fixed effects. Age was categorized as foals and weanlings (< 1 year), yearlings and adolescents (1–3 years), adults (4–18 years), and seniors (> 18 years) [42]. The same predictor structure was subsequently applied to the IFAT model to allow direct comparison between both diagnostic outcomes. To address separation in the PCR data, the PCR model was refitted using Firth-penalized logistic regression, retaining the same predictors and complete-case dataset. Model adequacy was assessed using simulation-based residual diagnostics implemented in the DHARMa package [43], including tests for residual uniformity, dispersion, and outliers. Multicollinearity was evaluated using variance inflation factors (VIF) calculated with the performance package [44]. The overall contribution of each predictor was assessed using likelihood-ratio tests comparing the full model with models excluding the respective factor. Regression coefficients were reported as estimates with standard errors, with two-sided penalized likelihood-ratio *P*-values for the PCR model and z-statistics, and two-sided Wald *P*-values for the IFAT model. For interpretation, coefficients were exponentiated and presented as adjusted odds ratios (OR) with 95% confidence intervals (CI). Model-adjusted pairwise comparisons among factor levels were evaluated using two-sided penalized likelihood-ratio tests for the PCR model and reported as pairwise ORs with 95% profile penalized likelihood CI and *P*-values adjusted using the Holm method within each predictor. For the IFAT model, pairwise ORs with 95% CI were calculated from estimated marginal means using the emmeans package [45], with Tukey-adjusted *P*-values. All possible pairwise comparisons were calculated, whereas forest plots were restricted to predictors with at least one statistically significant pairwise contrast. To investigate whether associations between horse type and test positivity varied by region, season, age category, sex, or sampling year, exploratory interaction analyses were performed. Each interaction was added separately to the corresponding model containing all main effects. Firth-penalized logistic regression was used for both PCR and IFAT interaction analyses to address separation in sparse subgroups. Interactions were evaluated using penalized likelihood-ratio tests, with Holm adjustment across the five tests for each diagnostic outcome.

## Results

### Study population

A total of 7,081 horses were included, which were tested by PCR and/or IFAT. Three-hundred-fifty-eight of 2753 (13.0%) horses were tested positive by PCR, and 1201/4615 (26.0%) by IFAT. Both PCR and IFAT were carried out on 287/7,081 horses (4.1%). Of these, 13/287 (4.5%) tested positive by PCR and IFAT, 25/287 (8.7%) by PCR only, and 97/287 (33.8%) by IFAT only. The remaining 152/287 horses (53%) tested negative in both PCR and serology.

Data on breed were available for 3893/7081 horses (55%, Table 1). The proportion of positive results varied across horse type classifications for both PCR and IFAT. PCR-positivity was highest in thoroughbreds (20.0%), followed by warmblood horses (16.5%), ponies (9.1%), and heavy horses (8.0%). In contrast, IFAT-seropositivity was highest in heavy horses (42.2%), followed by ponies (29.8%), thoroughbreds (27.4%), and warmblood horses (25.6%) (Table 1). Overall, 2208/5377 horses (41.1%) with known sex were tested by PCR and 3,412/5,377 (63.5%) by IFAT. Among PCR-tested horses, 205/1326 male horses (15.5%), including 49/317 intact males (15.5%) and 156/1,009 geldings (15.5%), and 94/882 female horses (10.7%) were tested positive. IFAT-positivity was observed in 500/1881 male horses (26.6%), including 108/473 intact males (22.8%) and 392/1,408 geldings (27.8%), and 410/1531 female horses (26.8%).

**Table 1:** Distribution of PCR and IFAT results according to horse type, in Anaplasma *phagocytophilum* polymerase chain reaction (PCR) and/or immunofluorescence antibody test (IFAT) results in 3893 horses (*n* positive/*N* total (%, 95%-confidence interval))

Horse type	*n* positive/*N* total (%)	PCR	IFAT
Warmblood horses	2,594/3,893 (66.6)	186/1,124 (16.5, 14.5–18.8)	410/1,602 (25.6, 23.5–27.8)
Thoroughbreds	226/3,893 (5.8)	26/130 (20.0, 14.0–27.7)	29/106 (27.4, 19.8–36.5)
Heavy horses	132/3,893 (3.4)	6/75 (8.0, 3.7–16.4)	27/64 (42.2, 30.9–54.4)
Ponies	941/3,893 (24.2)	40/441 (9.1, 6.7–12.1)	162/544 (29.8, 26.1–33.8)
Total	3,893/3,893 (100)	258/1,770 (14.6, 13.0–16.3)	628/2,316 (27.1, 25.3–29.0)

**Fig. 1:**
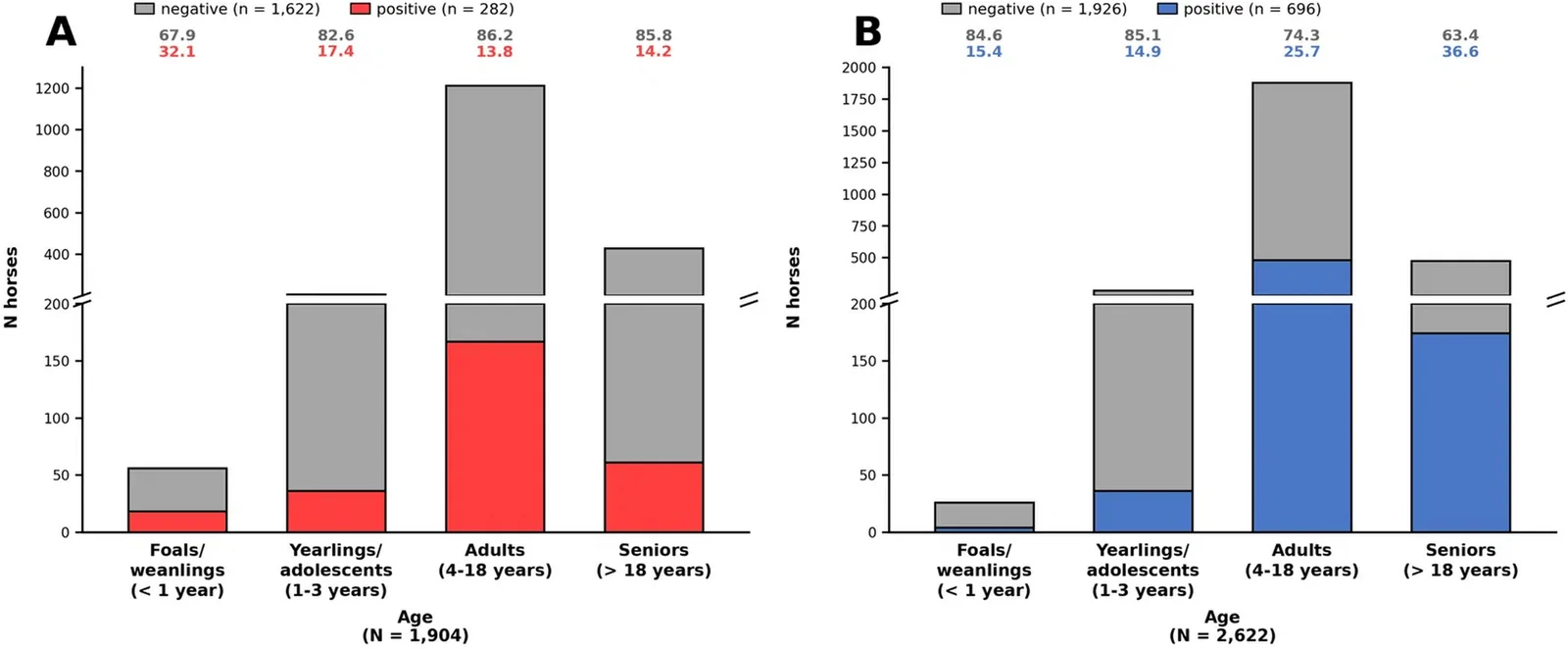
Age distribution (in age categories) of *Anaplasma phagocytophilum* polymerase chain reaction (PCR) (**A**) and immunofluorescence antibody test (IFAT) (**B**) results. Numbers above the bars represent the percentage of horses tested positive (colored) and negative (grey)

The age was known for 4315/7081 (60.9%) horses, with a mean of 12 years, a median of 11 years and a range of two months to 38 years. PCR-positivity was detected in 18/56 (32.1%) foals and weanlings, 36/207 (17.4%) yearlings and adolescents, 61/429 (14.2%) seniors, and 167/1212 (13.8%) adults (Fig. 1). IFAT-positivity was observed in 4/26 (15.4%) foals and weanlings, 36/242 (14.9%) yearlings and adolescents, 482/1878 (25.7%) adults, and 174/476 (36.6%) seniors (Fig. 1).

### Seasonality

**Fig. 2:**
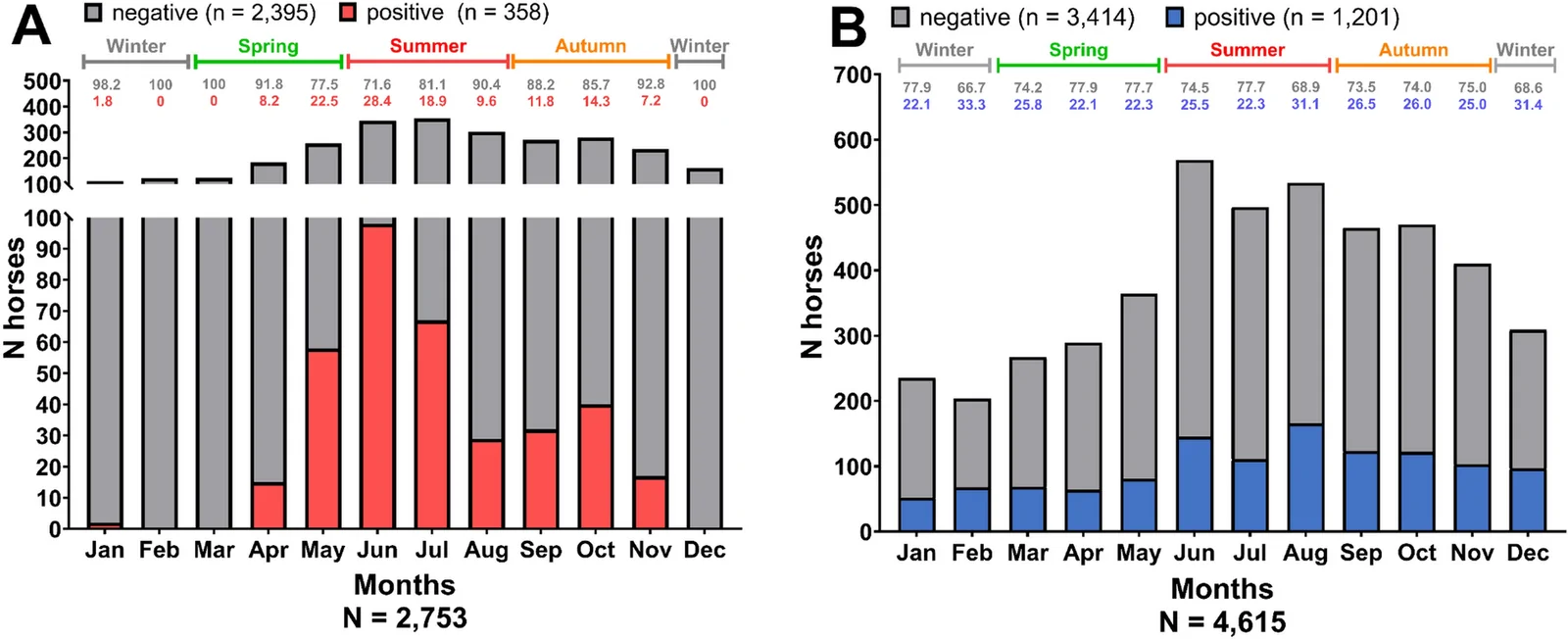
Monthly distribution of *Anaplasma phagocytophilum* polymerase chain reaction (PCR) (**A**) and immunofluorescence antibody test (IFAT) (**B**) results. Numbers above the bars represent the percentage of horses tested positive (colored) and negative (grey)

The date of sampling and testing was known for all 7081 horses. Seasonal differences were observed for both PCR and IFAT results. PCR-positivity was highest in summer (19.3%), followed by spring (12.9%), autumn (11.3%), and winter (0.5%) (Table 2, Fig. 2, Suppl. Table 1). In contrast, IFAT-positivity was highest in winter (29.0%), followed by summer (26.4%), autumn (25.8%), and spring (23.3%) (Table 2, Fig. 2, Suppl. Table 1).

**Table 2:** Seasonal distribution of horses tested by *Anaplasma phagocytophilum* polymerase chain reaction (PCR) and/or immunofluorescence antibody test (IFAT) in Europe (*n* positive/*N* total (%, 95% confidence interval))

Season	PCR	IFAT
Spring^1^	73/566 (12.9, 10.4–15.9)	214/920 (23.3, 20.7–26.1)
Summer^2^	194/1.003 (19.3, 17.0–21.9)	422/1,600 (26.4, 24.3–28.6)
Autumn^3^	89/787 (11.3, 9.3–13.7)	348/1,347 (25.8, 23.6–28.2)
Winter^4^	2/397 (0.5, 0.1–1.8)	217/748 (29.0, 25.9–32.4)
Total	358/2,753 (13.0, 11.8–14.3)	1201/4,615 (26.0, 24.8–27.3)

^1^Spring: March–May, ^2^summer: June–August, ^3^autumn: September–November, ^4^winter: December–February

### Regional distribution

**Fig. 3:**
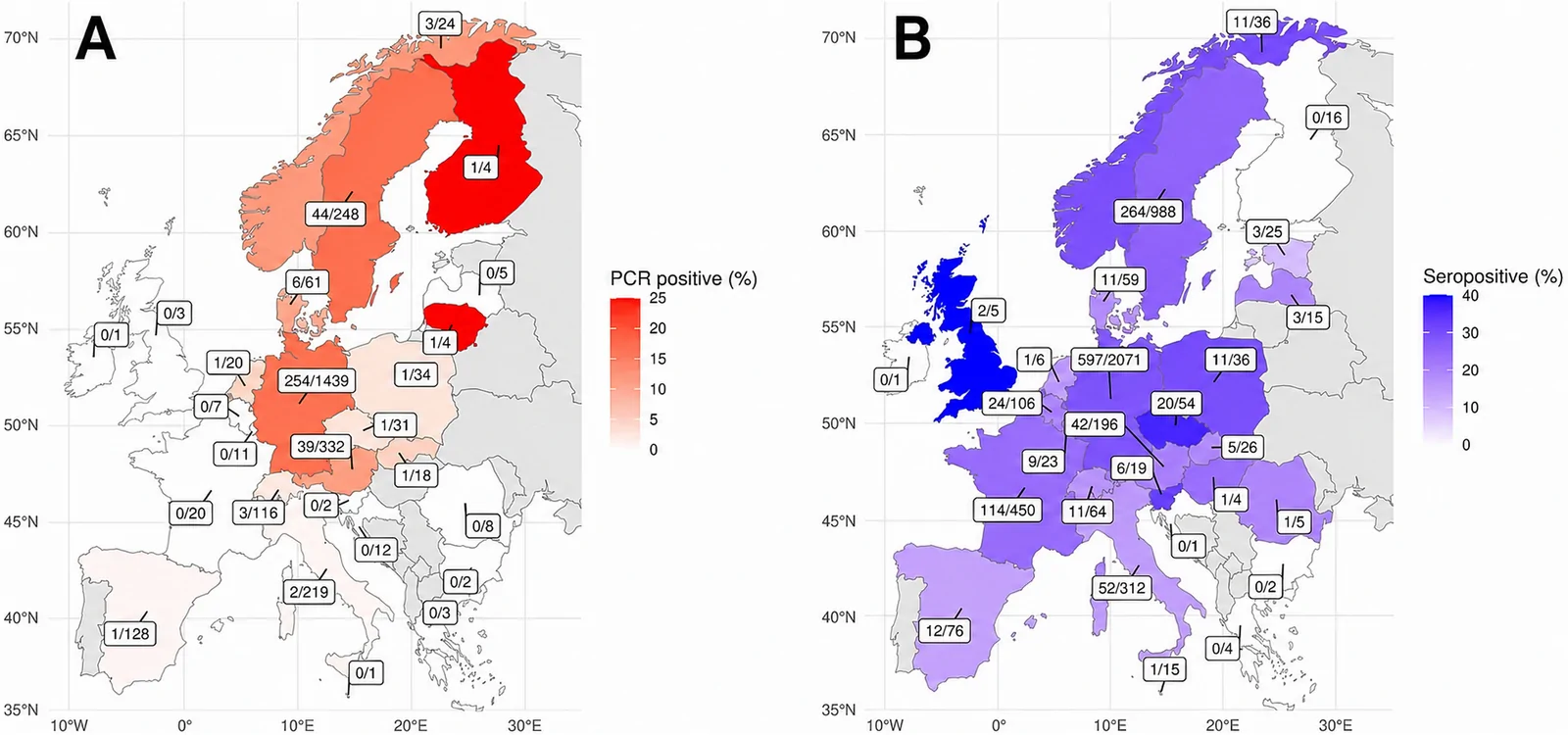
Geographic distribution of *Anaplasma phagocytophilum* test results in horses (number of positives/total number of samples) by European country, based on polymerase chain reaction (PCR) testing (n = 2753) (**A**) and immunofluorescence antibody testing (IFAT) (n = 4615) (**B**). Note that sample numbers for some countries are low

Information on the country of origin was available for all 7081 horses included in the study. PCR-positivity differed across European regions, with the highest proportions observed in North (15.8%) and Central (15.7%), followed by markedly lower values in East (4.0%), West (1.7%), and South (0.8%) (Table 3). In contrast, IFAT-positivity showed a more homogeneous distribution across most European regions, with similarly high proportions in Central (27.9%), East (26.7%), North (26.1%), and West (25.3%), while South (16.6%) exhibited a lower seroprevalence (Table 3). The geographical distribution of PCR and IFAT results across European countries is shown in Fig. 3.

**Table 3:** Regional distribution of horses tested by *Anaplasma phagocytophilum* polymerase chain reaction (PCR) and/or immunofluorescence antibody test (IFAT) in Europe (*n* positive/*N* total (%, 95% confidence interval))

European region	PCR	IFAT
Central^1^	296/1,887 (15.7, 14.1–17.4)	650/2,331 (27.9, 26.1–29.7)
East^2^	4/100 (4.0, 1.6–9.8)	44/165 (26.7, 20.5–33.9)
West^3^	1/58 (1.7, 0.3–9.1)	148/585 (25.3, 22.0–29.0)
North^4^	54/341 (15.8, 12.3–20.1)	288/1,105 (26.1, 23.6–28.7)
South^5^	3/367 (0.8, 0.3–2.4)	71/429 (16.6, 13.3–20.4)
Total	358/2,753 (13.0, 11.8–14.3)	1,201/4,615 (26.0, 24.8–27.3)

^1^Central: Austria, Germany, Switzerland; ^2^East: Czech Republic, Estonia, Hungary, Lithuania, Latvia, Poland, Romania, Slovakia; ^3^West: Belgium, France, Luxembourg, Netherlands; ^4^North: Denmark, Finland, United Kingdom, Ireland, Norway, Sweden; ^5^South: Bulgaria, Spain, Greece, Croatia, Italy, Malta, Slovenia

### Years of testing

Over the study period, the proportion of PCR-positive horses was 14.2% in 2022, 11.8% in 2023, 10.7% in 2024, and 14.9% in 2025. The percentages of horses tested serologically positive was 20.3% in 2022, 24.0% in 2023, 27.4% in 2024, and 29.4% in 2025. More detailed results are given in Table 4.

**Table 4:** Anapla*sma phagocytophilum*-polymerase chain reaction and immunofluorescence antibody test (IFAT) results in horses from Europe from 2022 to 2025 (*n* positive/*N* total (%, 95% confidence interval))

Year	PCR	IFAT
2022	67/471 (14.2, 11.4–17.7)	174/857 (20.3, 17.8–23.1)
2023	67/568 (11.8, 9.4–14.7)	236/985 (24.0, 21.4–26.7)
2024	80/748 (10.7, 8.7–13.1)	326/1,189 (27.4, 25.0–30.0)
2025	144/966 (14.9, 12.8–17.3)	465/1,584 (29.4, 27.2–31.7)
Total	358/2,753 (13.0, 11.8–14.3)	1,201/4,615 (26.0, 24.8–27.3)

### Multiple logistic regression analysis and adjusted pairwise comparisons

The final model for PCR- and IFAT-positivity included 1417 and 1748 horses, respectively, corresponding to 51.5% of 2753 PCR-tested horses and 37.9% of 4615 IFAT-tested horses. The remaining horses were excluded from the respective multivariable models because of missing predictor data (complete-case analysis). Within-category comparisons of positivity proportions between horses included in and excluded from the complete-case analysis revealed higher PCR positivity among included horses sampled in spring (17.6% versus 8.4%, *P* = 0.026), whereas no other comparisons reached statistical significance for either PCR or IFAT positivity (Suppl. Table 2). Residual diagnostics for the PCR and IFAT models indicated no significant deviations from the expected residual distribution for either model. All VIF values were <2, indicating no relevant multicollinearity among the included predictors.

### PCR model

In the PCR model, global penalized likelihood-ratio tests indicated significant associations between PCR positivity and all predictors included in the model. Season made the strongest overall contribution to model fit (χ^2^ = 63.64, df = 3, *P* < 0.001), followed by European region (*χ*^2^ = 18.20, df = 4, *P* = 0.001). Significant overall effects were also observed for horse type classification (χ^2^ = 12.12, df = 3, *P* = 0.007), age category (χ^2^ = 9.70, df = 3, *P* = 0.021), sampling year (χ^2^ = 9.45, df = 3, *P* = 0.024), and sex (χ^2^ = 7.47, df = 2, *P* = 0.024).

**Fig. 4:**
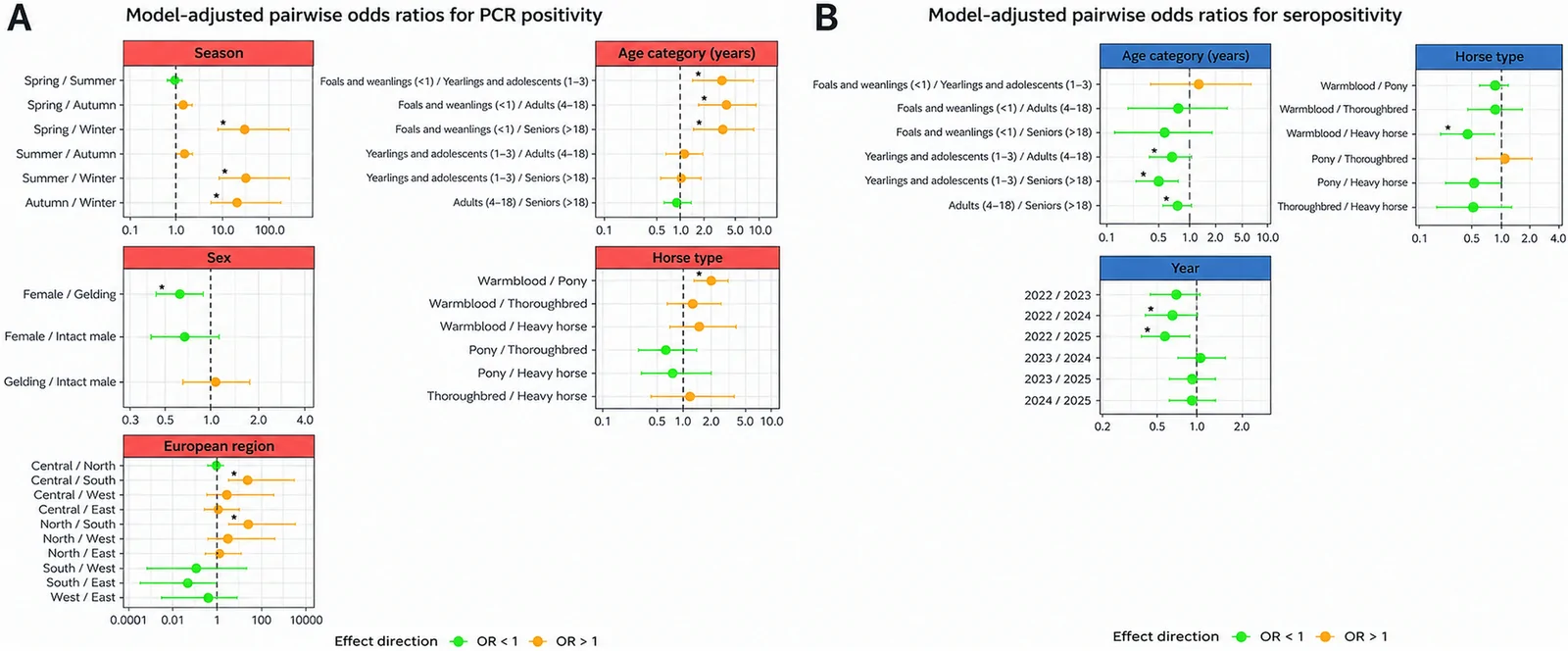
Model-adjusted pairwise odds ratios for positive *Anaplasma phagocytophilum* test results in horses in Europe from 2022 to 2025, based on polymerase chain reaction (PCR) testing (n = 1417) (**A**) and immunofluorescence antibody testing (IFAT) (n = 1748) (**B**). PCR odds ratios (OR) were derived from the Firth-penalized logistic regression model, whereas IFAT odds were derived from estimated marginal means based on the conventional logistic regression model. Error bars indicate 95% confidence intervals. Pairwise comparisons are shown for predictors with at least one statistically significant contrast. The dashed vertical line indicates an OR of 1. The asterisk (*) indicates a statistically significant pairwise comparison (adjusted *P* < 0.05)

The clearest effect was observed for season. Compared with spring, which served as the reference category, winter was associated with lower odds of PCR-positivity (OR = 0.03, *P* < 0.001) (Table 5). Adjusted pairwise comparisons confirmed significantly higher odds of PCR-positivity in horses tested in spring, summer, and autumn than horses tested in winter, whereas no significant differences were detected among spring, summer and autumn (Fig. 4, Suppl. Table 3).

**Table 5:** Firth-penalized logistic regression analysis in 1417 horses tested by means of *Anaplasma phagocytophilum* polymerase chain reaction (PCR) in Europe from 2022 to 2025.

	Estimate	SE^1^	Adjusted OR^2^	95% CI^3^ for OR		*P*-value
				Lower bound	Upper bound	
Sex (intact male)	− 0.070	0.248	0.933	0.564	1.510	0.780
Sex (female)	− 0.466	0.176	0.628	0.441	0.887	0.008*
Age category foals and weanlings (< 1 year)	1.368	0.429	3.927	1.666	9.236	0.002*
Age category yearlings and adolescents (1–3 years)	0.133	0.265	1.142	0.663	1.906	0.624
Age category seniors (> 18 years)	0.094	0.193	1.099	0.745	1.602	0.629
Year 2023	− 0.400	0.256	0.670	0.403	1.113	0.122
Year 2024	− 0.615	0.247	0.541	0.331	0.884	0.014*
Year 2025	− 0.082	0.217	0.921	0.602	1.427	0.709
Horse type (pony)	− 0.688	0.208	0.502	0.328	0.750	<0.001*
Horse type (thoroughbred)	− 0.222	0.351	0.801	0.383	1.571	0.530
Horse type (heavy horse)	− 0.399	0.427	0.671	0.261	1.475	0.339
Season (summer^5^)	0.066	0.193	1.068	0.733	1.574	0.733
Season (autumn^6^)	− 0.358	0.223	0.699	0.449	1.088	0.113
Season (winter^7^)	− 3.421	0.812	0.033	0.004	0.123	<0.001*
Region (Northern Europe^8^)	0.132	0.229	1.142	0.716	1.775	0.570
Region (Western Europe^9^)	− 1.090	1.428	0.336	0.003	2.737	0.379
Region (Eastern Europe^10^)	− 0.209	0.868	0.811	0.087	3.545	0.808
Region (Southern Europe^11^)	− 3.216	1.395	0.040	0.0003	0.286	<0.001*

^1^SE: standard error on the log-odds ratio scale; ^2^OR: odds ratio; CI^3^: confidence interval; ^4^spring: March–May, ^5^summer: June–August, ^6^autumn: September–November, ^7^winter: December–February; ^8^North: Denmark, Finland, United Kingdom, Ireland, Norway, Sweden; ^9^West: Belgium, France, Luxembourg, Netherlands; ^10^East: Czech Republic, Estonia, Hungary, Lithuania, Latvia, Poland, Romania, Slovakia; ^11^South: Bulgaria, Spain, Greece, Croatia, Italy, Malta, Slovenia; ^12^Central: Austria, Germany, Switzerland

Reference categories: sex (gelding); age category adults (4–18 years); year 2022; horse type (warmblood); season (spring^4^); Region (central Europe^12^)

Significant *P*-values are marked with an asterisk

Age-related differences were primarily attributable to higher odds of PCR-positivity for foals and weanlings. Compared with adult horses, which constituted the reference category, foals and weanlings showed higher odds in the PCR regression model (OR = 3.93, *P* = 0.002) (Table 5). Pairwise comparisons confirmed higher odds for foals and weanlings than for adults, yearlings and adolescents, and seniors, while no further significant pairwise differences were observed (Fig. 4, Suppl. Table 3).

Sex and horse type classification was also associated with PCR-positivity. Female horses had lower odds than geldings, as indicated by both the regression coefficient (OR = 0.63, *P* = 0.008) (Table 5) and pairwise comparison (Fig. 4, Suppl. Table 3). No significant differences were detected between geldings and intact males or between females and intact males (Fig. 4, Suppl. Table 3). Regarding horse type classification, ponies had lower odds of PCR-positivity than warmbloods, which served as the reference category (OR = 0.50, *P*<0.001) (Table 5). Accordingly, the pairwise comparison showed higher odds for warmbloods than for ponies, while no other pairwise differences among horse type classifications were statistically significant (Fig. 4, Suppl. Table 3). Exploratory interaction analyses provided no statistical evidence of interactions between horse type and the other model predictors for PCR-positivity (Suppl. Table 4).

Regarding the sampling year, the regression coefficient indicated lower odds of PCR-positivity in 2024 than in the reference year 2022 (OR = 0.54, *P* = 0.014) (Table 5); however, none of the Holm-adjusted pairwise comparisons among sampling years reached statistical significance (Suppl. Table 3).

Model-adjusted pairwise comparisons indicated higher odds of PCR-positivity in Central and Northern Europe than in Southern Europe (OR = 24.932 and 28.460, respectively; both *P* < 0.001), whereas no other regional pairwise comparisons reached statistical significance (Fig. 4, Suppl. Table 3). Regional effect sizes remained imprecisely estimated, as reflected by wide confidence intervals, particularly for comparisons involving Southern and Western Europe, where data were sparse and no PCR-positive horses remained in the complete-case dataset (0/65 and 0/12, respectively; cf. Suppl. Table 2).

### IFAT model

In the IFAT model, global likelihood-ratio tests indicated significant associations of seropositivity with age category (χ^2^ = 26.36, df = 3, *P* < 0.001), sampling year (χ^2^ = 14.05, df = 3, *P* = 0.003), and horse type classification (χ^2^ = 13.50, df = 3, *P* = 0.004). No significant overall effects were observed for season (χ^2^ = 4.54, df = 3, *P* = 0.209), European region (χ^2^ = 4.30, df = 4, *P* = 0.367) or sex (χ^2^ = 0.53, df = 2, *P* = 0.768).

Age-related differences were mainly driven by higher odds of seropositivity in older horses. Compared with adult horses, which constituted the reference category, seniors had higher odds of seropositivity (OR = 1.64, *P* < 0.001), whereas yearlings and adolescents had lower odds (OR = 0.46, *P* = 0.003) (Table 6). Pairwise comparisons confirmed higher odds for seniors than for adults and yearlings and adolescents, and higher odds for adults than for yearlings and adolescents (Fig. 4, Suppl. Table 3).

**Table 6:** Multiple logistic regression analysis in 1748 horses tested by means of Anaplasma phagocytophilum immunofluorescence antibody test (IFAT) in Europe from 2022 to 2025

	Estimate	SE^1^	z-value	Adjusted OR^2^	95% CI^3^ for OR		***P***-value
					Lower bound	Upper bound	
Sex (intact male)	− 0.119	0.199	− 0.601	0.888	0.597	1.301	0.548
Sex (female)	0.024	0.117	0.203	1.024	0.814	1.288	0.839
Age category foals and weanlings (< 1 year)	− 0.584	0.653	− 0.894	0.558	0.126	1.776	0.371
Age category yearlings and adolescents (1–3 years)	− 0.776	0.261	− 2.977	0.460	0.269	0.750	0.003*
Age category seniors (> 18 years)	0.495	0.139	3.565	1.640	1.248	2.152	<0.001*
Year 2023	0.413	0.173	2.380	1.510	1.077	2.126	0.0174*
Year 2024	0.436	0.167	2.620	1.548	1.118	2.150	0.009*
Year 2025	0.573	0.158	3.621	1.772	1.304	2.425	<0.001*
Horse type (pony)	0.251	0.130	1.930	1.290	0.994	1.656	0.054
Horse type (thoroughbred)	0.226	0.284	0.795	1.250	0.704	2.158	0.427
Horse type (heavy horse)	1.007	0.298	3.377	2.740	1.519	4.921	<0.001*
Season (summer^5^)	0.231	0.156	1.479	1.259	0.929	1.714	0.139
Season (autumn^6^)	0.168	0.161	1.045	1.183	0.865	1.623	0.296
Season (winter^7^)	0.369	0.179	2.060	1.446	1.018	2.056	0.039*
Region (Northern Europe^8^)	− 0.071	0.134	− 0.527	0.932	0.715	1.210	0.598
Region (Western Europe^9^)	0.079	0.288	0.275	1.082	0.605	1.884	0.783
Region (Eastern Europe^10^)	− 0.044	0.366	− 0.120	0.957	0.448	1.908	0.905
Region (Southern Europe^11^)	− 0.544	0.283	− 1.923	0.580	0.324	0.987	0.055

^1^SE: standard error on the log-odds ratio scale; ^2^OR: odds ratio; CI^3^: confidence interval. ^4^spring: March–May, ^5^summer: June–August, ^6^autumn: September–November, ^7^winter: December–February; ^8^North: Denmark, Finland, United Kingdom, Ireland, Norway, Sweden; ^9^West: Belgium, France, Luxembourg, Netherlands; ^10^East: Czech Republic, Estonia, Hungary, Lithuania, Latvia, Poland, Romania, Slovakia; ^11^South: Bulgaria, Spain, Greece, Croatia, Italy, Malta, Slovenia; ^12^Central: Austria, Germany, Switzerland

Reference categories: sex (gelding); age category adults (4–18 years); year 2022; Horse type (warmblood); season (spring^4^); Region (central Europe^12^)

Significant *P*-values are marked with an asterisk

Regarding horse type classification, heavy horses had higher odds of seropositivity than warmbloods, which served as the reference category (OR = 2.74, *P* < 0.001) (Table 6). This difference was confirmed by pairwise comparison, whereas no other pairwise differences among horse type classifications were statistically significant (Fig. 4, Suppl. Table 3). As in the PCR analyses, no statistically significant interactions were detected between horse type and the other predictors for IFAT seropositivity (Suppl. Table 4).

Sampling year had a significant overall effect on seropositivity. Compared with the reference year 2022, horses sampled in 2023 (OR = 1.51, *P* = 0.017), 2024 (OR = 1.55, *P* = 0.009), and 2025 (OR = 1.77, *P* < 0.001) showed higher odds of seropositivity (Table 6). Pairwise comparisons confirmed significantly higher odds in 2024 and 2025 than in 2022, while the remaining pairwise contrasts among sampling years were not statistically significant (Fig. 4, Suppl. Table 3).

Although the regression coefficient for winter indicated higher odds of seropositivity than in spring, season was not significant in the global likelihood-ratio test, and none of the Tukey-adjusted pairwise comparisons among seasons reached statistical significance (Table 6, Suppl. Table 3). Thus, there was no strong evidence for a meaningful seasonal effect on seropositivity. Similarly, neither sex nor European region showed significant overall effects or significant pairwise contrasts (Table 6, Suppl. Table 3).

## Discussion

*Anaplasma phagocytophilum* is a zoonotic pathogen infecting almost all mammals and humans [46], and is therefore important regarding the One-Health aspect and All-species approach. The pathogen was detected by PCR in 13.0% of horses in the present study, and antibodies were detected in 26.0% by IFAT. PCR remains the method of choice for confirming acute *A. phagocytophilum* infection, as it allows direct detection of the pathogen [33, 47]. It should be noted that PCR positivity reflects detectable *A. phagocytophilum* DNA at the time of testing and, in the absence of individual clinical information, may not correspond to clinically manifest acute EGA in all positive horses [32]. In contrast, a single serological result is not reliable for diagnosing infection due to delayed seroconversion and antibody persistence. Therefore, serological testing is suitable for assessing pathogen contact in the past and mainly of epidemiological significance but of limited clinical value [48].

Dogs show generally lower percentages of positive *A. phagocytophilum* PCR results in Europe compared to the 13.0% of horses testing PCR-positive in the present study (Germany 6.3% [49], Italy 0% [50], Poland 1% [51], Portugal 0% [52], United Kingdom 0.8% [53]). A similar pattern can be observed in cats across Europe with PCR-positivity reported at 7.5%; however, data in cats remains much more limited [38]. Horses included in this study were most likely preselected by veterinarians based on clinical suspicion of EGA, which may influence the observed PCR-positivity rate, but the same is true for dogs and cats in most of the referenced studies. Horses may have a higher risk of tick exposure compared to dogs, cats, and humans, resulting in higher PCR-positivity. Horses are often kept on pastures for prolonged periods, facilitating contact with tick vectors over a longer period. Additionally, products licensed against ticks are not available for horses.

On the other hand, seropositivity is comparable between horses, dogs and cats, indicating a similar exposure to *A. phagocytophilum* (dogs: Germany 43.2% [49], Italy 8.8–32.8% [54, 55], Portugal 55% [52], Spain 11.5% [56], Sweden 17.7% [57], Switzerland 7.5% [58]; cats: Germany 9.1–23% [3, 59, 60], Italy 4.5–33.3% [61, 62], Spain 1.8–8.4% [63–66], Switzerland 24% [3], Sweden 22.1% [67]). Data were compared with dogs and cats because a broader data set of prevalence is available for both species, allowing a reasonable epidemiological comparison with horses. However, tick exposure or ectoparasite control practices are not directly comparable between the species. The discrepancy between comparable seropositivity across species but different PCR-positivity rates may reflect differences in clinical awareness and testing practices between species rather than differences in exposure. Furthermore, species-specific differences in the pathogenesis of *A. phagocytophilum* infection may also contribute to the higher PCR-positivity observed in horses. Nevertheless, differences in study design, geographical distribution, sampling strategies, and diagnostic methods between published studies may also have contributed to the observed variation in PCR-positivity.

In the present study, a pronounced seasonal pattern was observed for *A. phagocytophilum* PCR-positivity in horses. Pairwise comparisons showed that horses tested in spring, summer, or autumn all had significantly higher odds of PCR-positivity than horses tested in winter. Importantly, no significant differences in PCR-positivity were detected among spring, summer, and autumn, indicating that the elevated risk extends across the entire season with pronounced *I. ricinus* activity. The seasonal distribution observed in this study closely follows the known activity pattern of *I. ricinus*. Tick activity is typically low during winter, increases in spring with peaks in late spring and early summer, and may show a second peak during autumn depending on climatic conditions [68, 69]. Direct pathogen detection by PCR reflects the presence of circulating pathogen DNA during the bacteremic phase and therefore closely mirrors periods of increased tick exposure. Besides DNA present in the blood, clinical signs of EGA may not be evident as subclinical and self-limiting infections are described in horses [32]. In contrast, serological results showed weaker seasonality, which can be explained by the persistence of antibodies after the acute phase of infection [48]. Similar discrepancies between PCR- and serology-based seasonality have been reported in previous equine studies [15] as well as in canines [2]. Nevertheless, sporadic PCR positive cases detected outside the main tick season indicate that EGA should be considered as a differential diagnosis all year round. As mentioned above, *I. ricinus* remains active during mild winter periods, and ongoing climate change may further extend the seasonal activity of ticks, potentially increasing the risk of EGA also during winter months [70].

In the present data, PCR-positivity for *A. phagocytophilum* in horses was associated with European region, with significantly higher odds in Central and Northern Europe than in Southern Europe. No regional pairwise contrast reached statistical significance in the IFAT model. These findings require cautious interpretation because the complete-case PCR dataset contained no positive horses from Southern or Western Europe (0/65 and 0/12, respectively), although positive horses were present in the full diagnostic dataset (3/367 and 1/58, respectively). Although Firth regression analysis addresses separation, the sparse regional data, particularly the small sample size for Western Europe, limited the precision of the estimates. The wide confidence intervals indicate uncertainty about the magnitude of the regional differences. The descriptive data showed higher PCR-positivity in Northern (15.8%) and Central Europe (15.7%) compared to Eastern (4.0%), Western (1.7%), and Southern Europe (0.8%). While this pattern broadly aligns with the geographical distribution of *I. ricinus*, which thrives in temperate climates with high vegetation density and sufficient moisture [71], it should not be interpreted as evidence for a generally higher tick abundance or reservoir host density in northern Europe compared to other regions. Instead, differences in diagnostic approaches, sampling strategies, and awareness are likely to contribute substantially to the observed variation. Published prevalence estimates in horses vary considerably between studies and regions. In Sweden, PCR-positivity ranges from 17–69%, while seroprevalence (IFAT) is reported at 17%, indicating a discrepancy between direct pathogen detection and evidence of exposure [10, 11, 72]. This contrast may reflect differences in sampling strategies, such as preferential testing of clinically suspect cases, as well as a higher level of awareness and targeted diagnostics in regions where the pathogen was first described. In comparison, central European countries, including the Czech Republic (PCR: 5%; IFAT: 5–73%) [17, 19], the Netherlands (PCR: 10%) [13], Switzerland (IFAT: 4%) [21], and Germany (PCR: 15%; IFAT: 27%) [15], generally report lower and more variable prevalence estimates, while studies from southern European countries, such as Portugal (PCR: 4.3%) [73] and Greece (IFAT: 14.2%) [29], tend to show lower values overall.

The observed increase in seropositivity from 2022 to 2025 does not necessarily indicate a true rise in pathogen circulation. It can also be explained by increased awareness among veterinarians over the study period and a consequently higher frequency of targeted diagnostic testing, particularly in febrile horses.

Age was significantly associated with seropositivity. Older horses showed higher odds of being seropositive, most likely due to cumulative exposure to infected ticks over their lifetime. Similar age-related increases in seroprevalence have been reported previously in equine and canine studies and are generally interpreted as a marker of repeated or long-term exposure rather than increased susceptibility to EGA infection [2, 15]. In addition to higher seropositivity in senior horses, foals and weanlings showed higher odds of PCR positivity compared to adult horses in the present study. This may reflect age-related differences in immune function, as immune responses in foals are not yet fully mature during the first weeks to months of life [74].

Female horses had lower odds of testing PCR positive compared to geldings; no significant difference was observed between geldings and intact males or between females and intact males. Although the biological basis for this observation remains unclear, differences in management practices, housing conditions, pasture access, or usage patterns (e.g., sport versus leisure horses) may contribute to differential tick exposure. Testosterone has been proposed to modulate host–tick interactions in some species [39, 40, 75], although our findings do not support a clear role for testosterone in infection risk, as intact males did not differ significantly from either geldings or females. Further studies are needed to clarify the sex-related differences in PCR-positivity observed here.

Horse type-associated differences were observed for PCR-positivity, with ponies showing significantly lower odds compared to warmbloods, and for seropositivity, with heavy horses showing significantly higher odds compared to warmbloods. The observed differences, even after adjustment for European region, season, age category, sex, and sampling year, were an unexpected finding. Exploratory interaction models, investigating whether the associations between horse type and test positivity varied across categories of the other model predictors, detected no statistically significant interactions. Although an association with the measured sampling characteristics cannot be excluded, particularly because statistical power may have been limited within individual subgroups, the lower odds observed in ponies and the higher odds observed in heavy horses may instead reflect unmeasured differences in husbandry, management, and exposure to tick habitats.

Limitations of our retrospective study are lacking information on housing, management practices, and pasture access, all of which may affect tick exposure and infection risk. Samples were submitted to a diagnostic laboratory, and the reasons for testing were unknown, indicating preselection by veterinarians based on clinical suspicion or routine screening, which may have introduced selection bias. Regional assignment was based on veterinary postal codes rather than the horses’ actual locations or travel history. Due to the nature of our study, we were not able to screen the whole equine population across each country in detail, but were limited to the location of the veterinarian submitting the samples. However, categorization into European countries and broader regions likely minimizes major bias. Finally, uneven regional sample sizes, particularly the low number of samples from Western Europe, may have limited regional comparisons. Consequently, the reported PCR- and IFAT-positivity proportions should be interpreted as positivity among horses submitted for diagnostic testing rather than as prevalence estimates for the general European equine population. In addition, for horses with repeated testing, retaining the first positive sample rather than a uniform first-available sample may have introduced outcome-dependent selection with respect to season, sampling years, and age. This should be considered when interpreting the reported associations. Furthermore, multivariable analyses were based on complete-case analysis, which excluded a substantial proportion of horses with missing predictor data. The analysed subset may therefore differ systematically from the full diagnostic population.

## Conclusions

*Anaplasma phagocytophilum* DNA was detected in 13.0% of horses submitted for PCR testing. Twenty-six percent of horses tested by IFAT were seropositive, indicating past pathogen contact. *Anaplasma phagocytophilum* detection was widespread across Europe, underlining its relevance as a tick-borne pathogen in horses submitted for diagnostic testing. Model results indicated potential regional differences in PCR positivity as well as marked seasonal patterns, which is in accordance with the distribution of *I. ricinus* ticks in Europe and tick activity. EGA should nevertheless be considered as a differential diagnosis throughout the year. Increasing age was significantly associated with seropositivity, indicating cumulative lifetime exposure. Sex- and horse type-associated differences in PCR and IFAT results were identified and may reflect unmeasured differences in management, housing, and pasture exposure rather than intrinsic biological susceptibility. Compared with the limited published data available, horses showed higher PCR positivity than dogs and cats, whereas seropositivity rates appeared broadly comparable across species. Overall, there should be a year-round clinical awareness of EGA in Europe.

## Supplementary Information

Additional file 1. Monthly distribution of horses tested by *Anaplasma phagocytophilum* polymerase chain reactionand/or immunofluorescence antibody testin Europe).

Additional file 2. Comparison of *Anaplasma phagocytophilum* polymerase chain reactionand immunofluorescence antibody testpositivity between horses included in and excluded from the complete-case multivariable analyses in Europe, within each category of the model predictors).

Additional file 3. Model-adjusted pairwise comparisons for 1417 horses tested for *Anaplasma phagocytophilum* by polymerase chain reaction and 1748 horses tested by immunofluorescence antibody test in Europe.

Additional file 4. Exploratory analyses of interactions between horse type and other model predictors for 1417 horses tested for *Anaplasma phagocytophilum* polymerase chain reactionpositivity and 1748 horses tested by immunofluorescence antibody testpositivity in Europe.

## Data Availability

All data generated or analyzed during this study are included in the published article and its supplementary information files.

## Abbreviations

AIC: Akaike's information criterion
BIC: Bayesian information criterion
CI: Confidence interval
DNA: Deoxyribonucleic acid
EDTA: Ethylenediaminetetraacetic acid
EGA: Equine granulocytic anaplasmosis
ELISA: Enzyme-linked immunosorbent assay
HSP60: 60-KDa heat shock protein
IFAT: Immunofluorescence antibody test
OR: Odds ratio
PCR: Polymerase chain reaction
Suppl.: Supplementary
VIF: Variance inflation factors
