# Supplementary material for: Factors associated with Anaplasma phagocytophilum PCR positivity and seropositivity in horses in Europe (2022–2025)
Source: Parasit Vectors. 2026 Nov 14;19:494. doi: 10.1186/s13071-026-07770-x (PMC13770334; doi:10.1186/s13071-026-07770-x)
Supplement: Supplementary file 1 [file 13071_2026_7770_MOESM1_ESM.docx]

**Supplementary Table 1:** Monthly distribution of horses tested by *Anaplasma phagocytophilum* polymerase chain reaction (PCR) and/or immunofluorescence antibody test (IFAT) in Europe (*n* positive/*N* total (%, 95% confidence interval))

| **Month** | **PCR** | **IFAT** |
| --- | --- | --- |
| January | 2/112 (1.8, 0.5-6.3) | 52/235 (22.1, 17.3-27.9) |
| February | 0/123 (0, 0-3.0) | 68/204 (33.3, 27.2-40.1) |
| March | 0/124 (0, 0-3.0) | 69/267 (25.8, 21.0-31.4) |
| April | 15/184 (8.2, 5.0-13.0) | 64/289 (22.1, 17.7-27.3) |
| Mai | 58/258 (22.5, 17.8-28.0) | 81/364 (22.3, 18.3-26.8) |
| June | 98/345 (28.4, 23.9-33.4) | 145/569 (25.5, 22.1-29.2) |
| July | 67/355 (18.9, 15.1-23.3) | 111/497 (22.3, 18.9-26.2) |
| August | 29/303 (9.6, 6.7-13.4) | 166/534 (31.1, 27.3-35.1) |
| September | 32/271 (11.8, 8.5-16.2) | 123/465 (26.5, 22.7-30.6) |
| October | 40/280 (14.3, 10.7-18.9) | 122/470 (26.0, 22.2-30.1) |
| November | 17/236 (7.2, 4.5-11.2) | 103/410 (25.0, 21.2-29.5) |
| December | 0/162 (0, 0-2.3) | 97/309 (31.4, 26.5-36.8) |
| Total | 358/2,753 (13.0, 11.8-14.3) | 1,201/4,615 (26.0, 24.8-27.3) |
