# Supplementary material for: Factors associated with Anaplasma phagocytophilum PCR positivity and seropositivity in horses in Europe (2022–2025)
Source: Parasit Vectors. 2026 Nov 14;19:494. doi: 10.1186/s13071-026-07770-x (PMC13770334; doi:10.1186/s13071-026-07770-x)
Supplement: Supplementary file 2 [file 13071_2026_7770_MOESM2_ESM.docx]

**Supplementary Table 2:** Comparison of *Anaplasma phagocytophilum* polymerase chain reaction (PCR) and immunofluorescence antibody test (IFAT) positivity between horses included in and excluded from the complete-case multivariable analyses in Europe, within each category of the model predictors (*n* positive/*N* tested (%)). Raw and Holm-adjusted *P*-values refer to the comparison of positivity proportions between included and excluded horses within each category. The asterisk (*) indicates a statistically significant difference (adjusted *P* < 0.05).

| **Category** | **All tested** | **Included (complete-case)** | **Excluded** | ***P*-value (raw)** | ***P*-value (Holm)** |
| --- | --- | --- | --- | --- | --- |
| **Dataset for PCR model** | | | | | |
| Sex: gelding | 156/1009 (15.5%) | 114/675 (16.9%) | 42/334 (12.6%) | 0.079 | 1.000 |
| Sex: intact male | 49/317 (15.5%) | 37/196 (18.9%) | 12/121 (9.9%) | 0.037 | 0.823 |
| Sex: female | 94/882 (10.7%) | 61/546 (11.2%) | 33/336 (9.8%) | 0.575 | 1.000 |
| Age: foals and weanlings (<1 year) | 18/56 (32.1%) | 17/45 (37.8%) | 1/11 (9.1%) | 0.084 | 1.000 |
| Age: yearlings and adolescents (1-3 years) | 36/207 (17.4%) | 22/148 (14.9%) | 14/59 (23.7%) | 0.155 | 1.000 |
| Age: adults (4-18 years) | 167/1212 (13.8%) | 127/905 (14.0%) | 40/307 (13.0%) | 0.702 | 1.000 |
| Age: seniors (>18 years) | 61/429 (14.2%) | 46/319 (14.4%) | 15/110 (13.6%) | 1.000 | 1.000 |
| Year: 2022 | 67/471 (14.2%) | 39/222 (17.6%) | 28/249 (11.2%) | 0.064 | 1.000 |
| Year: 2023 | 67/568 (11.8%) | 37/278 (13.3%) | 30/290 (10.3%) | 0.299 | 1.000 |
| Year: 2024 | 80/748 (10.7%) | 45/390 (11.5%) | 35/358 (9.8%) | 0.478 | 1.000 |
| Year: 2025 | 144/966 (14.9%) | 91/527 (17.3%) | 53/439 (12.1%) | 0.029 | 0.672 |
| Horse type: Warmblood | 186/1124 (16.5%) | 155/910 (17.0%) | 31/214 (14.5%) | 0.414 | 1.000 |
| Horse type: Pony | 40/441 (9.1%) | 32/347 (9.2%) | 8/94 (8.5%) | 1.000 | 1.000 |
| Horse type: Thoroughbred | 26/130 (20.0%) | 19/102 (18.6%) | 7/28 (25.0%) | 0.437 | 1.000 |
| Horse type: Heavy horse | 6/75 (8.0%) | 6/58 (10.3%) | 0/17 (0.0%) | 0.327 | 1.000 |
| Season: Spring^1^ | 73/566 (12.9%) | 49/279 (17.6%) | 24/287 (8.4%) | 0.001 | 0.026* |
| Season: Summer^2^ | 194/1003 (19.3%) | 114/546 (20.9%) | 80/457 (17.5%) | 0.199 | 1.000 |
| Season: Autumn^3^ | 89/787 (11.3%) | 48/376 (12.8%) | 41/411 (10.0%) | 0.260 | 1.000 |
| Season: Winter^4^ | 2/397 (0.5%) | 1/216 (0.5%) | 1/181 (0.6%) | 1.000 | 1.000 |
| European region: Central^5^ | 296/1887 (15.7%) | 182/1152 (15.8%) | 114/735 (15.5%) | 0.897 | 1.000 |
| European region: North^6^ | 54/341 (15.8%) | 29/172 (16.9%) | 25/169 (14.8%) | 0.657 | 1.000 |
| European region: West^7^ | 1/58 (1.7%) | 0/12 (0.0%) | 1/46 (2.2%) | 1.000 | 1.000 |
| European region: East^8^ | 4/100 (4.0%) | 1/16 (6.2%) | 3/84 (3.6%) | 0.508 | 1.000 |
| European region: South^9^ | 3/367 (0.8%) | 0/65 (0.0%) | 3/302 (1.0%) | 1.000 | 1.000 |
| **Dataset for IFAT model** | | | | | |
| Sex: gelding | 392/1408 (27.8%) | 229/804 (28.5%) | 163/604 (27.0%) | 0.548 | 1.000 |
| Sex: intact male | 108/473 (22.8%) | 44/195 (22.6%) | 64/278 (23.0%) | 1.000 | 1.000 |
| Sex: female | 410/1531 (26.8%) | 210/749 (28.0%) | 200/782 (25.6%) | 0.299 | 1.000 |
| Age: foals and weanlings (<1 year) | 4/26 (15.4%) | 3/17 (17.6%) | 1/9 (11.1%) | 1.000 | 1.000 |
| Age: yearlings and adolescents (1-3 years) | 36/242 (14.9%) | 19/137 (13.9%) | 17/105 (16.2%) | 0.716 | 1.000 |
| Age: adults (4-18 years) | 482/1878 (25.7%) | 337/1269 (26.6%) | 145/609 (23.8%) | 0.215 | 1.000 |
| Age: seniors (>18 years) | 174/476 (36.6%) | 124/325 (38.2%) | 50/151 (33.1%) | 0.308 | 1.000 |
| Year: 2022 | 174/857 (20.3%) | 84/394 (21.3%) | 90/463 (19.4%) | 0.497 | 1.000 |
| Year: 2023 | 236/985 (24.0%) | 102/371 (27.5%) | 134/614 (21.8%) | 0.045 | 1.000 |
| Year: 2024 | 326/1189 (27.4%) | 124/423 (29.3%) | 202/766 (26.4%) | 0.278 | 1.000 |
| Year: 2025 | 465/1584 (29.4%) | 173/560 (30.9%) | 292/1024 (28.5%) | 0.327 | 1.000 |
| Horse type: Warmblood | 410/1602 (25.6%) | 309/1222 (25.3%) | 101/380 (26.6%) | 0.638 | 1.000 |
| Horse type: Pony | 162/544 (29.8%) | 130/407 (31.9%) | 32/137 (23.4%) | 0.066 | 1.000 |
| Horse type: Thoroughbred | 29/106 (27.4%) | 20/69 (29.0%) | 9/37 (24.3%) | 0.655 | 1.000 |
| Horse type: Heavy horse | 27/64 (42.2%) | 24/50 (48.0%) | 3/14 (21.4%) | 0.125 | 1.000 |
| Season: Spring^1^ | 214/920 (23.3%) | 94/374 (25.1%) | 120/546 (22.0%) | 0.267 | 1.000 |
| Season: Summer^2^ | 422/1600 (26.4%) | 163/584 (27.9%) | 259/1016 (25.5%) | 0.290 | 1.000 |
| Season: Autumn^3^ | 348/1347 (25.8%) | 137/506 (27.1%) | 211/841 (25.1%) | 0.441 | 1.000 |
| Season: Winter^4^ | 217/748 (29.0%) | 89/284 (31.3%) | 128/464 (27.6%) | 0.281 | 1.000 |
| European region: Central^5^ | 650/2331 (27.9%) | 316/1071 (29.5%) | 334/1260 (26.5%) | 0.115 | 1.000 |
| European region: North^6^ | 288/1105 (26.1%) | 118/474 (24.9%) | 170/631 (26.9%) | 0.447 | 1.000 |
| European region: West^7^ | 148/585 (25.3%) | 21/64 (32.8%) | 127/521 (24.4%) | 0.170 | 1.000 |
| European region: East^8^ | 44/165 (26.7%) | 11/44 (25.0%) | 33/121 (27.3%) | 0.844 | 1.000 |
| European region: South^9^ | 71/429 (16.6%) | 17/95 (17.9%) | 54/334 (16.2%) | 0.754 | 1.000 |

^1^spring: March–May, ^2^summer: June–August, ^3^autumn: September–November, ^4^winter: December–February; ^5^Central: Austria, Germany, Switzerland; ^6^North: Denmark, Finland, United Kingdom, Ireland, Norway, Sweden; ^7^West: Belgium, France, Luxembourg, Netherlands; ^8^East: Czech Republic, Estonia, Hungary, Lithuania, Latvia, Poland, Romania, Slovakia; ^9^South: Bulgaria, Spain, Greece, Croatia, Italy, Malta, Slovenia
