# Supplementary material for: Factors associated with Anaplasma phagocytophilum PCR positivity and seropositivity in horses in Europe (2022–2025)
Source: Parasit Vectors. 2026 Nov 14;19:494. doi: 10.1186/s13071-026-07770-x (PMC13770334; doi:10.1186/s13071-026-07770-x)
Supplement: Supplementary file 3 [file 13071_2026_7770_MOESM3_ESM.docx]

**Supplementary Table 3:** Model-adjusted pairwise comparisons for 1,417 horses tested for *Anaplasma phagocytophilum* by polymerase chain reaction (PCR) and 1,748 horses tested by immunofluorescence antibody test (IFAT) in Europe. The asterisk (*) indicates a statistically significant pairwise comparison (adjusted *P* < 0.05)

|  | **OR^1^** | **SE^2^** | ***P*-value** |
| --- | --- | --- | --- |
| **PCR** | | | |
| Spring^3^ / summer^4^ | 0.936 | 0.180 | 0.733 |
| Spring / autumn^5^ | 1.431 | 0.319 | 0.225 |
| Spring / winter^6^ | 30.605 | 24.863 | <0.001* |
| Summer / autumn | 1.529 | 0.289 | 0.073 |
| Summer / winter | 32.701 | 26.283 | <0.001* |
| Autumn / winter | 21.392 | 17.363 | <0.001* |
| Year 2022 / year 2023 | 1.492 | 0.382 | 0.487 |
| Year 2022 / year 2024 | 1.849 | 0.457 | 0.072 |
| Year 2022 / year 2025 | 1.086 | 0.236 | 0.781 |
| Year 2023 / year 2024 | 1.239 | 0.306 | 0.781 |
| Year 2023 / year 2025 | 0.728 | 0.159 | 0.487 |
| Year 2024 / year 2025 | 0.587 | 0.120 | 0.052 |
| Central^7^ / North^8^ | 0.876 | 0.201 | 1.000 |
| Central / South^9^ | 24.932 | 34.779 | <0.001* |
| Central / West^10^ | 2.975 | 4.248 | 1.000 |
| Central / East^11^ | 1.233 | 1.071 | 1.000 |
| North / South | 28.460 | 40.099 | <0.001* |
| North / West | 3.396 | 4.895 | 1.000 |
| North / East | 1.407 | 1.251 | 1.000 |
| South / West | 0.119 | 0.238 | 1.000 |
| South / East | 0.049 | 0.081 | 0.403 |
| West / East | 0.414 | 0.690 | 1.000 |
| Adults (4-18 years) / foals and weanlings (<1 year) | 0.255 | 0.109 | 0.012* |
| Adults (4-18 years) / yearlings and adolescents (1-3 years) | 0.876 | 0.232 | 1.000 |
| Adults (4-18 years) / seniors (>18 years) | 0.910 | 0.176 | 1.000 |
| Foals and weanlings (<1 year) / yearlings and adolescents (1-3 years) | 3.439 | 1.547 | 0.028* |
| Foals and weanlings (<1 year) / seniors (>18 years) | 3.573 | 1.607 | 0.028* |
| Yearlings and adolescents (1-3 years) / seniors (>18 years) | 1.039 | 0.313 | 1.000 |
| Gelding / intact male | 1.072 | 0.266 | 0.780 |
| Gelding / female | 1.593 | 0.281 | 0.025* |
| Intact male / female | 1.486 | 0.373 | 0.249 |
| Warmblood / pony | 1.991 | 0.414 | 0.004* |
| Warmblood / thoroughbred | 1.248 | 0.438 | 1.000 |
| Warmblood / heavy horse | 1.490 | 0.637 | 1.000 |
| Pony / thoroughbred | 0.627 | 0.243 | 1.000 |
| Pony / heavy horse | 0.748 | 0.343 | 1.000 |
| Thoroughbred / heavy horse | 1.193 | 0.640 | 1.000 |
| **IFAT** | | | |
| Spring / summer | 0.794 | 0.124 | 0.451 |
| Spring / autumn | 0.846 | 0.136 | 0.723 |
| Spring / winter | 0.691 | 0.124 | 0.166 |
| Summer / autumn | 1.065 | 0.148 | 0.969 |
| Summer / winter | 0.871 | 0.141 | 0.829 |
| Autumn / winter | 0.818 | 0.136 | 0.620 |
| Year 2022 / year 2023 | 0.662 | 0.115 | 0.081 |
| Year 2022 / year 2024 | 0.646 | 0.108 | 0.044* |
| Year 2022 / year 2025 | 0.564 | 0.089 | 0.002* |
| Year 2023 / year 2024 | 0.976 | 0.158 | 0.999 |
| Year 2023 / year 2025 | 0.852 | 0.131 | 0.727 |
| Year 2024 / year 2025 | 0.873 | 0.127 | 0.786 |
| Central / North | 1.073 | 0.144 | 0.985 |
| Central / South | 1.723 | 0.487 | 0.305 |
| Central / West | 0.924 | 0.266 | 0.999 |
| Central / East | 1.045 | 0.383 | 1.000 |
| North / South | 1.605 | 0.476 | 0.501 |
| North / West | 0.861 | 0.259 | 0.987 |
| North / East | 0.973 | 0.365 | 1.000 |
| South / West | 0.536 | 0.209 | 0.500 |
| South / East | 0.606 | 0.273 | 0.801 |
| West / East | 1.131 | 0.511 | 0.999 |
| Adults (4-18 years) / foals and weanlings (<1 year) | 1.792 | 1.170 | 0.808 |
| Adults (4-18 years) / yearlings and adolescents (1-3 years) | 2.172 | 0.566 | 0.015* |
| Adults (4-18 years) / seniors (>18 years) | 0.610 | 0.085 | 0.002* |
| Foals and weanlings (<1 year) / yearlings and adolescents (1-3 years) | 1.212 | 0.840 | 0.993 |
| Foals and weanlings (<1 year) / seniors (>18 years) | 0.340 | 0.225 | 0.360 |
| Yearlings and adolescents (1-3 years) / seniors (>18 years) | 0.281 | 0.080 | <0.001* |
| Gelding / intact male | 1.127 | 0.224 | 0.820 |
| Gelding / female | 0.977 | 0.114 | 0.978 |
| Intact male / female | 0.867 | 0.172 | 0.751 |
| Warmblood / pony | 0.778 | 0.101 | 0.215 |
| Warmblood / thoroughbred | 0.798 | 0.227 | 0.857 |
| Warmblood / heavy horse | 0.365 | 0.109 | 0.004* |
| Pony / thoroughbred | 1.025 | 0.304 | 1.000 |
| Pony / heavy horse | 0.469 | 0.145 | 0.069 |
| Thoroughbred / heavy horse | 0.458 | 0.183 | 0.205 |

^1^OR: odds ratio; ^2^SE: standard error on the odds ratio scale; ^3^spring: March–May, ^4^summer: June–August, ^5^autumn: September–November, ^6^winter: December–February; ^7^Central: Austria, Germany, Switzerland; ^8^North: Denmark, Finland, United Kingdom, Ireland, Norway, Sweden; ^9^South: Bulgaria, Spain, Greece, Croatia, Italy, Malta, Slovenia; ^10^West: Belgium, France, Luxembourg, Netherlands; ^11^East: Czech Republic, Estonia, Hungary, Lithuania, Latvia, Poland, Romania, Slovakia
