# Supplementary material for: Factors associated with Anaplasma phagocytophilum PCR positivity and seropositivity in horses in Europe (2022–2025)
Source: Parasit Vectors. 2026 Nov 14;19:494. doi: 10.1186/s13071-026-07770-x (PMC13770334; doi:10.1186/s13071-026-07770-x)
Supplement: Supplementary file 4 [file 13071_2026_7770_MOESM4_ESM.docx]

**Supplementary Table 4:** Exploratory analyses of interactions between horse type and other model predictors for 1,417 horses tested for *Anaplasma phagocytophilum* polymerase chain reaction (PCR) positivity and 1,748 horses tested by immunofluorescence antibody test (IFAT) positivity in Europe. Each interaction term was added separately to the corresponding model containing all main effects.

|  |  | **PCR model** | | **IFAT model** | |
| --- | --- | --- | --- | --- | --- |
| **Interaction term** | **Df^1^** | **χ²^2^** | **Adjusted *P*-value** | **χ²^2^** | **Adjusted *P*-value** |
| Horse type x European region | 12 | 15.081 | 0.948 | 19.954 | 0.340 |
| Horse type x Season | 9 | 15.455 | 0.396 | 7.320 | 1.000 |
| Horse type x Age category | 9 | 4.739 | 1.000 | 9.627 | 1.000 |
| Horse type x Sex | 6 | 2.437 | 1.000 | 7.292 | 1.000 |
| Horse type x Sampling year | 9 | 5.120 | 1.000 | 10.165 | 1.000 |

^1^df: degrees of freedom; ^2^χ²: penalised likelihood-ratio test statistic
